# An Integrated View on Neuronal Subsets in the Peripheral Nervous System and Their Role in Immunoregulation

**DOI:** 10.3389/fimmu.2021.679055

**Published:** 2021-07-12

**Authors:** Manuel O. Jakob, Michael Kofoed-Branzk, Divija Deshpande, Shaira Murugan, Christoph S. N. Klose

**Affiliations:** ^1^ Department of Microbiology, Infectious Diseases and Immunology, Charité – Universitätsmedizin Berlin, Corporate Member of Freie Universität Berlin and Humboldt-Universität zu Berlin, Berlin, Germany; ^2^ Department of BioMedical Research, Group of Visceral Surgery and Medicine, University of Bern, Bern, Switzerland

**Keywords:** neuro-immune interactions, neuronal classification, peripheral nervous system, enteric nervous system, dorsal root ganglia (DRG), function of neurons

## Abstract

The peripheral nervous system consists of sensory circuits that respond to external and internal stimuli and effector circuits that adapt physiologic functions to environmental challenges. Identifying neurotransmitters and neuropeptides and the corresponding receptors on immune cells implies an essential role for the nervous system in regulating immune reactions. Vice versa, neurons express functional cytokine receptors to respond to inflammatory signals directly. Recent advances in single-cell and single-nuclei sequencing have provided an unprecedented depth in neuronal analysis and allowed to refine the classification of distinct neuronal subsets of the peripheral nervous system. Delineating the sensory and immunoregulatory capacity of different neuronal subsets could inform a better understanding of the response happening in tissues that coordinate physiologic functions, tissue homeostasis and immunity. Here, we summarize current subsets of peripheral neurons and discuss neuronal regulation of immune responses, focusing on neuro-immune interactions in the gastrointestinal tract. The nervous system as a central coordinator of immune reactions and tissue homeostasis may predispose for novel promising therapeutic approaches for a large variety of diseases including but not limited to chronic inflammation.

## Introduction

The nervous system in multi-cellular organisms consists of a complex network of neurons, which can rapidly and precisely transmit signals. Signal transmission within the nervous system is mediated by the release of neurotransmitters or neuropeptides from the presynaptic neuron into the synaptic cleft, the engagement of the cognate receptor on the postsynaptic neuron, and the elicitation of a signaling cascade within the postsynaptic neuron. The nervous system is organized in circuits linking afferent sensory input with a broad array of reactions at effector sites. In this way, the nervous system coordinates physiological functions, such as behavior, motor functions, blood pressure and hormone release (1–4). The nervous system is classified into the central nervous system (CNS) and the peripheral nervous system (PNS). The CNS, which includes the brain and the spinal cord, is enclosed by the dura mater. The PNS, placed outside of the dura mater, is sub-classified into the somatic and autonomic nervous systems. The somatic nervous system consists of peripheral somatosensory nerves, which convey afferent signals and efferent nerves controlling motor functions, e.g. regulating movements of extremities. The autonomic nervous system can be functionally distinguished into the sympathetic, the parasympathetic, and the enteric nervous system (5). The parasympathetic and sympathetic nervous system are typical functional counter players for opposing physiological functions: the rest-and-digest (parasympathetic nervous system) and the fight-or-flight reaction (sympathetic nervous system). These functions are highly conserved across species and build the basis for survival during external threats (6). The third component of the autonomic nervous system is the gut *intrinsic* enteric nervous system (ENS), which controls intestinal movement, mixing of ingested food and the secretion of fluids. In the gastrointestinal tract, *intrinsic* neurons are those whose cell bodies lie within the organ, whereas *extrinsic* nerves (e.g. sensory nerve fibers) have their cell bodies outside the innervated organ. Typically, the soma of extrinsic sensory afferents is located within dorsal root ganglia, celiac ganglia, superior or inferior mesenteric ganglia or the nodose/jugular ganglia. The intrinsic and independent coordinator ability of the ENS has been underlined in experiments following extrinsic denervation, where a lack of extrinsic signals only impaired physiologic functions of the intestine to a minor degree. Conversely, loss of the intrinsic ENS can be disastrous, as shown in Hirschsprung or Chagas disease, where intestinal motor functions are significantly reduced or absent (7, 8).

The ENS is composed of different neuronal populations, which fulfil specific physiologic functions. The traditional classification of neurons solely according to their chemical signature is not entirely sufficient to define a functional type of neuron because similar neurotransmitters seem to exert different physiologic functions. In the same line, anatomical/morphological classifications do not adhere to specific neuronal functions because of similar shapes of enteric neurons in distinct functional classes. Thus, current neuronal classifications need to be extended by a broader array of markers to better understand the peripheral nervous system in detail. Single-cell RNA-sequencing is a powerful tool, which provides several vital transcripts per cell and can zoom in at the potential correct resolution.

Here we review the current understanding of the function of different neuronal subsets that relay the signals to subsets of immune cells in the peripheral nervous system, which regulate intestinal physiology as a response to environmental challenges and physiologic perceptions.

## The Enteric Nervous System

The intestine has its own nervous system, the ENS, which operates to a large degree autonomously and outside of voluntary control, despite being innervated by extrinsic nerve fibers. The ENS is the largest collection of neurons outside the CNS comprising hundreds of millions of neurons. It is the major coordinator of physiologic bowel functions, including but not limited to peristaltic movement. The importance of the autonomous function of the ENS has been demonstrated by experiments using extrinsic denervation of the intestine, in which the bowel function was only mildly affected by the cessation of extrinsic signals (9).

The ENS has a characteristic spider web structure, is embedded in the intestinal wall and closely associated with the muscle layers. The somas of enteric neurons mainly cluster in two anatomically distinct but strongly interconnected ganglionated plexuses (10). The outer, myenteric plexus (Auerbach Plexus) lies within the longitudinal and the circular muscle layer, and the inner, submucosal plexus (Meissner Plexus) is located below the muscle layers (11).

While a classification based on morphological aspects has been proposed, this classification does not take into account that functionally similar neurons can vary in their morphology. Using open-end single-cell or single-nuclei sequencing approaches, several studies have revealed the transcriptomic landscape of the ENS and thus provide a classification, which is more functionally substantiated. A classification based on a transcriptional code, as discussed in the following paragraph, includes marker genes encoding for receptors, ion channels and neuropeptides and can thus broaden our understanding of physiologic and immunologic functions.

### Populations of Neurons

**Figure 1:**
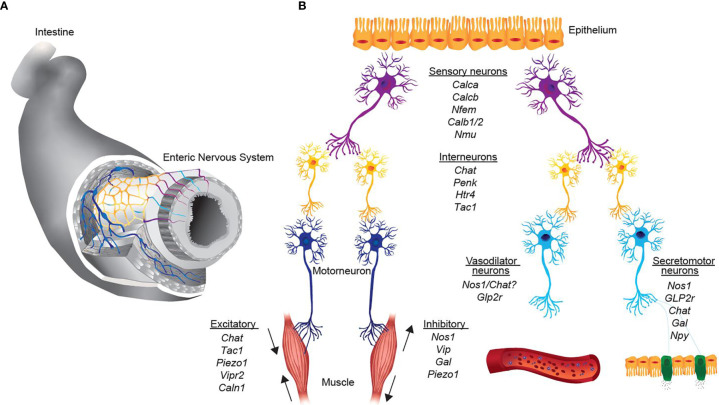
Representation of neurons, key transcripts and functional assignments in the intrinsic enteric nervous system. **(A)** 2-Dimensional scheme of the enteric nervous system. Colors represent the schematic representation of distinct functional neuronal groups. **(B)** Simplified representation from sensing of the environment of different stimuli (sensory neurons, purple), signaling transmission to interneurons (yellow) and excitation of effector neurons (motor neurons, dark blue; vasodilator and secretomotor, light blue). Italics are the key transcripts of the respective neuronal group.

The intrinsic micro-circuitry regulation of intestinal function consists of five neuronal classes with distinct functional specializations. Sensory neurons, also referred to as intrinsic primary afferent neurons (IPANs), detect chemical and physical alterations in the intestine and transmit the signal *via* interneurons to excitatory motor neurons, inhibitory motor neurons or secretomotor/vasodilator neurons (**Figure 1**). Enteric neurons are replenished from neuronal stem cells of the intestine. While the traditional sub-classification was based on the neuronal type, the anatomic location and axonal projection as well as neurochemical signature, recent advances in single-cell sequencing has enabled a comprehensive clustering based on gene expression.

Single-cell sequencing of the ENS using fluorescence-activated sorting of Wnt1-Cre; R26Tomato mice revealed 1105 enteric neurons (with ~ 3066 genes detected on average) and eventually found nine clusters of neurons in the muscular sheet of the intestine (12). Based on neuro-transmitters nitric oxide synthase 1 (*NOS1*) and choline acetyltransferase (*ChAT*), two main groups can be classified, *NOS1* ^+^ neurons and *ChAT* ^+^ neurons. The authors assigned three distinct neuronal clusters of nitrergic neurons, which comprise inhibitory motor neurons and secretomotor/vasodilator neurons (**Table 1**). Further, 6 clusters of cholinergic neurons, which express *ChAT* and the solute carrier family 5 member 7 (*Slc5a7*, gene encodes for an ion transporter) could be distinguished (12). *ChAT* ^+^ neurons are further subdivided into excitatory motor neurons, sensory neurons and interneurons according to their gene expression (**Table 1**).

**Table 1:** Reference genes for the identification of neuronal subsets based on unbiased single-cell RNA-sequencing (12–14).

Secretomotor, vasodilator	IMN		EMN		IN		SN		
NOS1			ChAT						
ENT1	ENT2	ENT3	ENT4	ENT5	ENT6	ENT7		ENT8	ENT9
**VIP low**	**VIP**	**VIP**	**Tac1**	**Tac1**	**Penk**	**Penk**		CCK	NMU
**Gal high**	**Gal**	**Gal**	**Piezo1**	**Piezo1**	**Htr4**	**Htr4**		Ucn3	Calcb
NeuroD6	**Piezo1**	**Piezo1**	**VIPr2**	**VIPr2**	**Tac1 high**	Calcb			Nog
Glp2r	NPY high	NPY low	**Caln1**	**Caln1**		Sst			

Bold genes represent key transcripts. IMN, inhibitory motor neurons; EMN, excitatory motor neurons; IN, Interneurons; SN, sensory neurons.

VIP, vasoactive intestinal peptide; Tac1, gene encoding Substance P; PENK, gene encoding proenkephalin (Opioid); Gal, galanin (neuroendocrine peptide); Piezo1, piezo type mechanosensitive ion channel component 1; Htr4, 5-hydroxytryptamine receptor 4; Caln1, Calneuron 1.

Using the pan-neuronal Baf53b-cre deleter mice crossed to R26R-tomato to enable sort-purification of enteric neurons from the myenteric plexus of the small intestine combined with single-cell RNA-sequencing, 4,892 high-quality enteric neurons have been analyzed and 12 classes of neurons could be identified based on their gene expression (13). Matching these results to known entities of enteric neurons based on functional properties, the authors could propose the following classification: Populations 1-4 can be classified as excitatory motor neurons predominantly defined *via* the expression of *Tac1* and *Calb2*. Class 8 and 9 have been assigned to inhibitory motor neurons and are characterized by the expression of *Nos1/Gal/VIP/Npy.* Different classes (Populations 6, 7, 11) of sensory intrinsic primary afferent neurons have been identified based on the known markers *Calca/Calcb/Nfem/Calb1/Calb2* and the selective expression for *NMU*, *Ucn-3/Cck* or *Nxph2*. Interneurons are represented with a mixed neurochemical signature, such as *Nos1*/*ChAT* for Interneurons group 1, *Sst/Calcb/Calb2* for interneurons group 2. The co-expression of *Nos1* and *ChAT* was also found in the dataset of Zeisel and, thus, may reflect the profile of interneurons, which connect different neuronal groups (12). While all the single-cell RNA-sequencing studies nicely delineate distinct populations of enteric neurons based on specific transcripts, dissociation and sort-purification of entire neurons from the muscular sheet of the intestine might not equally represent all neurons in the ENS, some of which might be sensitive to the isolation or sorting procedure. It should be noted that even for tissue-resident immune cells, which are presumably easier to release from the fabric of the tissue after digestion, a discrepancy between cells recovered after digestion and those detected in situ was reported (15).

To overcome this potential limitation, a study by the Regev lab performed single-nuclei sequencing by using sort-purification of labelled nuclei with a nuclear-tagged fluorescent protein (14). This approach is expected to result in an equal representation of neuronal nuclei present in the tissue. The authors sequenced 1´187´535 colonic and ileal nuclei and eventually profiled 2´657 neuronal nuclei (with 7´369 genes detected per nucleus). By using nuclear isolation and sequencing, Drokhlyansky and colleagues identified 21 neuronal populations based on known marker genes. These 21 identified neuronal classes could be further broadly sub-classified into 5 populations of *ChAT^+^Nos1^+^* double-expressing putative excitatory motor neurons, 7 populations of *Nos1^+^* inhibitory motor neurons (4 subsets are *Nos1^+^Vip^+^*), 2 populations of *Glp2r^+^* secretomotor and vasodilator neurons, 4 populations of *CGRP* ^+^ sensory neurons, 3 populations of *Penk^+^* Interneurons (**Table 1**). Apart from the transcripts at the single-cell level, there is limited data available that reports a deeper characterization of neuronal subsets after sort purification. Thus, the detection limit of single-cell RNA-sequencing can miss important transcripts in certain subpopulations of cells. Purification of neuronal subpopulations may allow to assign defined functions and to delineate the neuronal subclasses in more detail in the near future. In-between the above-mentioned studies, minor discrepancies in the representation of neuronal subclasses were reported, which may be due to different reporter mouse strains used for sort purification, different isolation/sequencing techniques or differences in bioinformatic analyses. However, even though transcripts may differ in between different single-cell RNA-sequencing datasets, the known functional subsets of enteric neurons are uniformly present in all studies. Consequently, each study concluded putative functional roles of identified neuronal subclasses by associating gene expression to a certain known function, even though without experimental proof. Thus, future studies need to experimentally confirm functional coherence of the identified transcripts.

Within the enteric nervous system, only one study performed sequencing of human neurons (14). By using MIRACL-sequencing, a total of 436'202 human nuclei were profiled and 1'445 neurons clustered into 14 neuronal subsets (with ~ 4302 genes detected on average). By comparing human and mouse colon neuronal nuclei, the authors found strong congruence between species but also differences in ENS composition, such as a higher abundance of motor neurons and a lower diversity of sensory neurons, interneurons and secretomotor/vasodilator neurons in humans. The fact that the abundance of neurons is relatively low, future studies should aim to address to enrich neurons in specimens to gain deeper insights in regulated neuronal gene expression.

## Expression of Genes Mediating Neuro-Immune Interaction in the ENS

**Figure 2:**
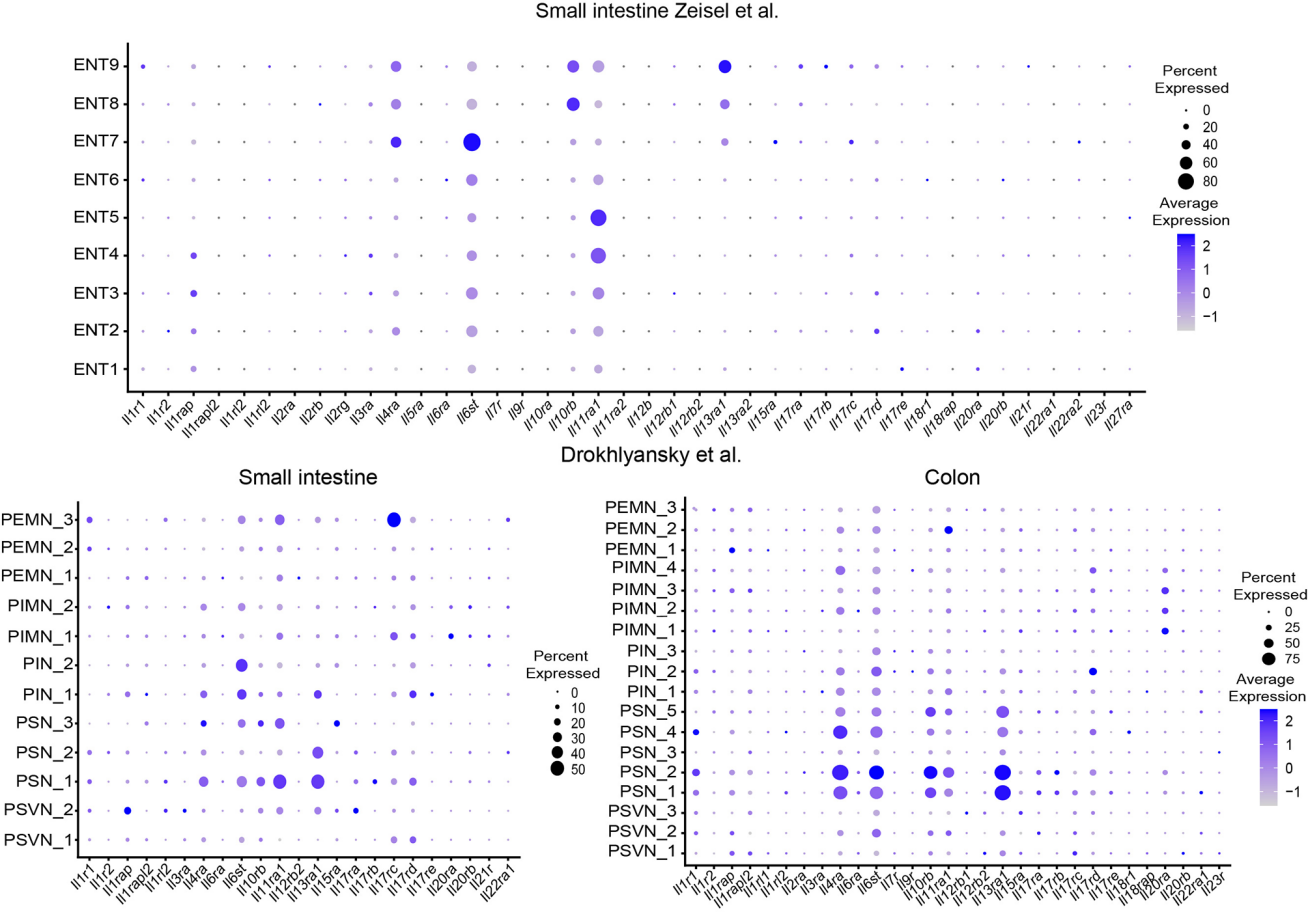
Expression of interleukin receptors in different ENS subsets. Dotplots showing the percentage of expressing cells as well as average expression within the indicated identified neuronal clusters for selected murine interleukin receptor genes. Data was downloaded from http://mousebrain.org/ (12) or from https://singlecell.broadinstitute.org/ (14). Genes not present in the plot showing the Drokhlyansky data have been filtered. All dataset were normalized and transformed before the plots were created using the Seurat package (16).

Understanding which genes enteric neurons have adopted for sensing the immune system is one of the burning questions in the field today. We aimed to provide an overview by re-examining published single-cell and single-nuclei datasets deposited in publicly available databases in silico for expression of cytokine receptors, chemokine receptors, NOD-like receptors (NLRs) and toll-like receptors (TLRs) (**Figures 2** **–** **4**) (12, 14). With regard to cytokine receptors *Il11ra1*, *Il4Ra*, *Il13ra1*, and *Il6st* (**Figure 2**) were detectable in enteric neurons.

The expression of the type 2 cytokine receptors *Il4Ra* and *IL13ra1* in sensory ganglia and their functional role has been studied in the context of chronic itch in mice and humans (17). Functionally, type 2 cytokines can directly activate sensory neurons, activate itch-sensory pathways and are critical players in the development of chronic itch sensations. Furthermore, type 2 cytokines induce the JAK signaling pathway in neurons and JAK inhibitors have been successfully tested in chronic itch diseases in humans (17). In the same line, the important initiator of type 2 responses thymic stromal lymphopetin (TSLP) activates TRPA1^+^ sensory neurons and promotes itch responses in mice (18). However, their role in the ENS needs to be delineated in future studies.

**Figure 3:**
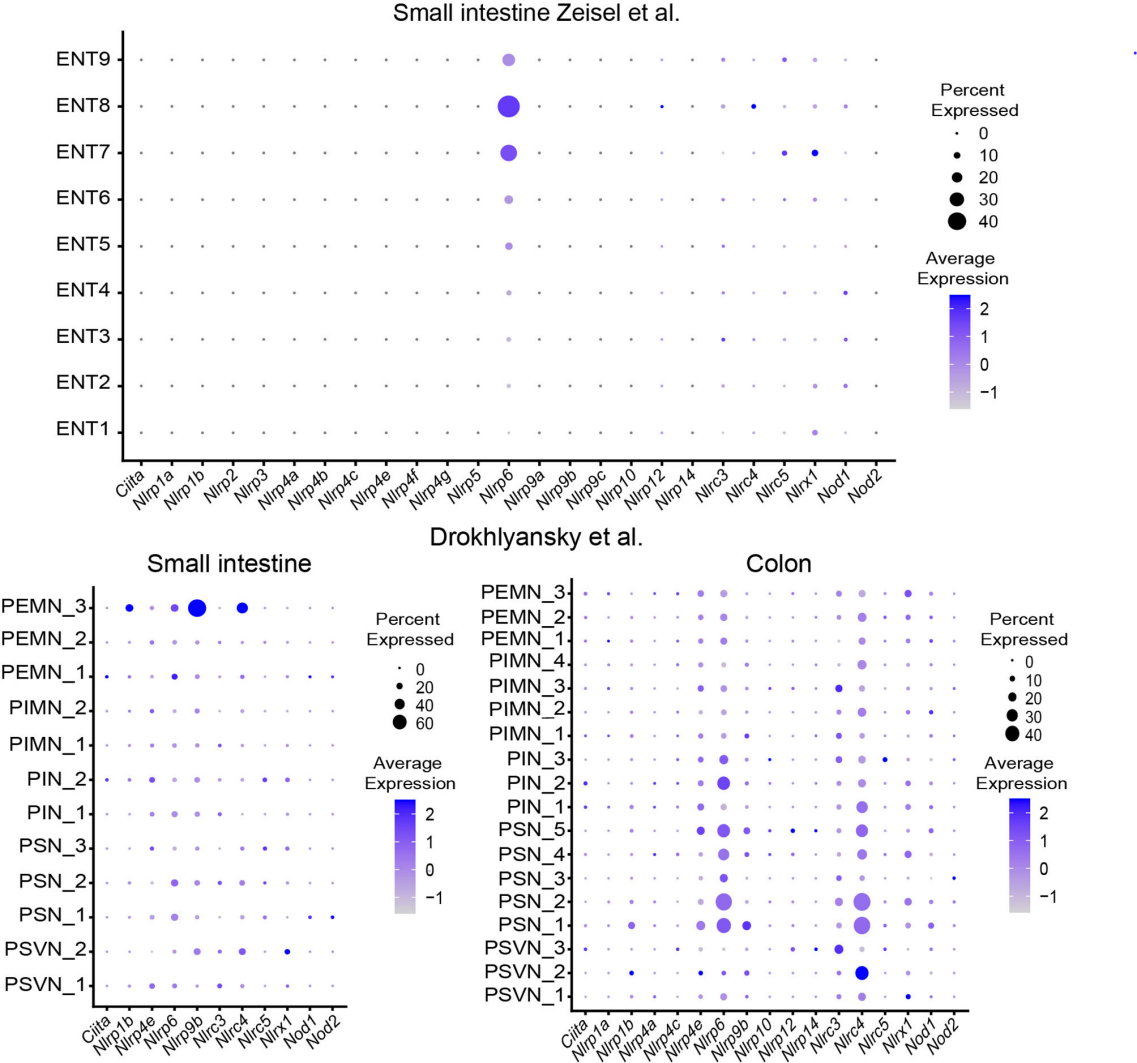
Expression of NOD-like receptors in different ENS subsets. Dotplots showing the percentage of expressing cells as well as average expression within the indicated identified neuronal clusters for selected murine NOD-like receptor genes. Data was downloaded from http://mousebrain.org/ (12) or from https://singlecell.broadinstitute.org/ (14). Genes not present in the plot showing the Drokhlyansky data have been filtered. All dataset were normalized and transformed before the plots were created using the Seurat package (16).

**Figure 4:**
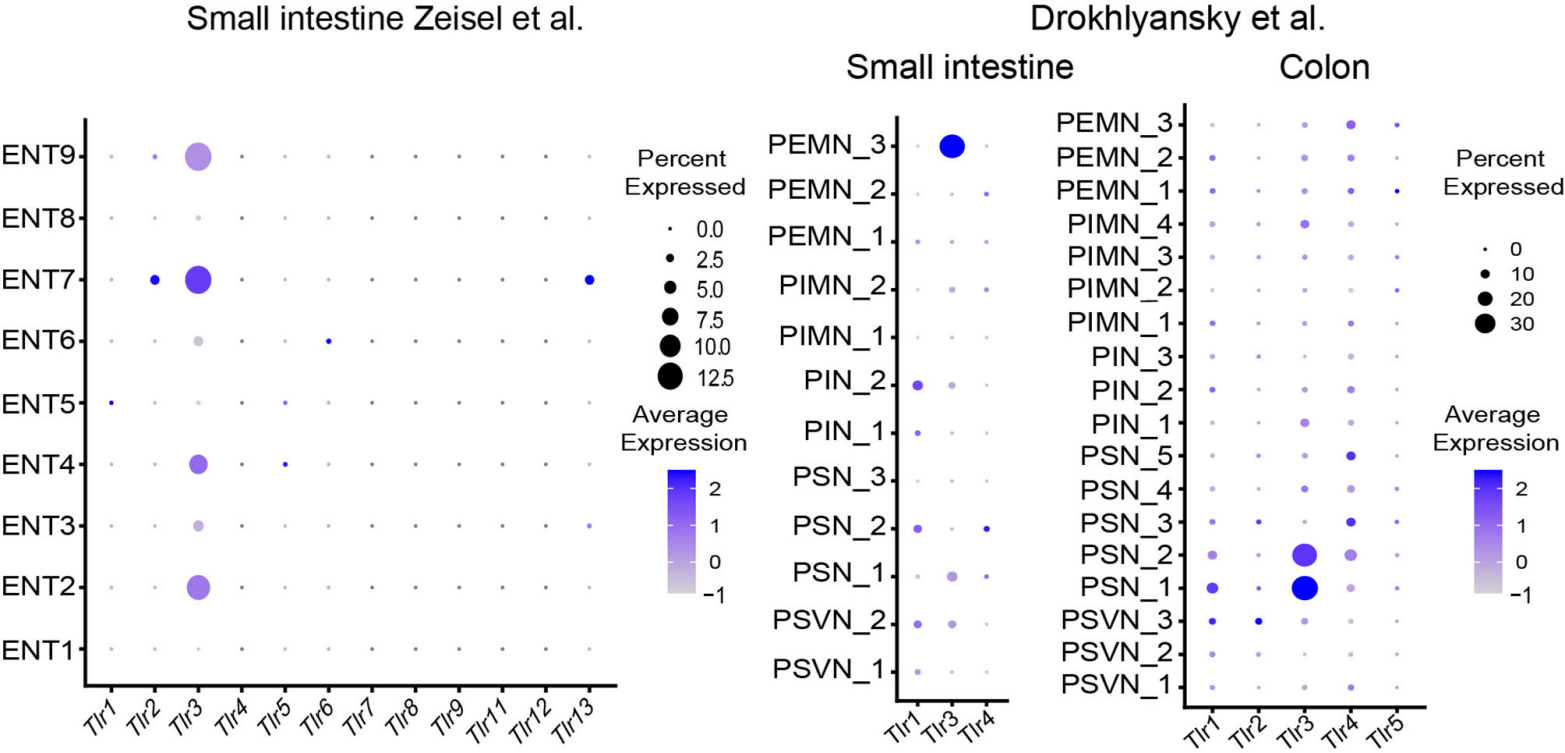
Expression of Toll-like receptors in different ENS subsets. Dotplots showing the percentage of expressing cells as well as average expression within the indicated identified neuronal clusters for selected murine Toll-like receptor genes. Data was downloaded from http://mousebrain.org/ (12) or from https://singlecell.broadinstitute.org/ (14). Genes not present in the plot showing the Drokhlyansky data have been filtered. All dataset were normalized and transformed before the plots were created using the Seurat package (16).

Recent studies suggest a role for pattern recognition receptors (PRR) in DRG neurons and pain sensation (19). Given its location, the ENS is constantly exposed to microbial factors. A role for the ENS in sensing microbial metabolites was found but the importance of PRR on enteric neurons is not well defined. Reanalysis of the datasets shows expression of *Nlrp6* and *TLR3* and is consistently found in enteric neurons among several studies (**Figures 3** and **4**).

The role of the inflammasome components Nlrp6 and caspase 11 has recently been highlighted to control enteric neuronal cell death and glucose metabolism in a microbiota-dependent manner (20). In fact, microbiota-depletion with antibiotics leads to loss of enteric associated CART^+^ neurons in a Nlrp6 and Caspase 11 dependent manner. Analysis of SPF-colonized and GF mice revealed reduction in blood glucose levels, which was linked to CART^+^ neurons suggesting the regulation of blood glucose level independent from CNS control (20). The role of TLRs in neurons is intriguing because it may suggest direct microbial sensing of enteric neurons (11). However, the role of TLR3 in the ENS remains elusive.

The expression pattern of cytokine receptors, NLRs and TLRs, and chemokine receptors can guide the readers to design their research projects accordingly (**Figures 2** **–** **4**, **Supplementary Figure 1**).

## Regulation of Neuronal Populations

### Excitatory and Inhibitory Motor Neurons

Within the group of motor neurons, a sub-classification proposed by Furness and colleagues (21) distinguishes five main classes of motor neurons present in the intestine including excitatory motor neurons, inhibitory motor neurons, secretomotor/vasodilator neurons, secretomotor neurons that are not vasodilator and neurons to enteroendocrine cells (**Figure 1**).

Excitatory motor neurons use the main neurotransmitter acetylcholine (Ach) for signal transmission and are thus distinguishable from inhibitory motor neurons, which use nitric oxide (NO) as the main neurotransmitter (5). This difference in signal transmission of excitatory versus inhibitory motor neurons becomes evident in anticholinergic medications, such as atropine or antidepressants, that typically lead to constipation due to the lack of signals from excitatory motor neurons (22). Furthermore, conditional deletion of ChAT in neural-crest derived neurons by using the Wnt1-cre driver in mice resulted in gastrointestinal dysmotility, dysbiosis and eventually death of the mice at post-natal day 30. This phenotype highlights the extraordinary role of *ChAT* for host physiology and its role in the coordination of motor functions (23).

Even though Ach is the dominant neurotransmitter used by excitatory motor neurons, these neurons show a residual excitation after muscarinic block suggesting that other neurotransmitters are involved (24, 25). The residual excitation is assumed to be mediated by tachykinins, in particular Substance P (gene *Tac1*), which engage on NK1 and NK2 receptors on muscle cells (26). Altogether, these data indicate that excitation of motor neurons is mediated by cholinergic and non-cholinergic neurotransmitters and each excitation may be fine-tuned dependent on the transmitter involved.

On the contrary, experiments in mice with the deletion of *NOS1* revealed that neurons normally expressing NOS remain intact (apart from grossly enlarged stomach due to pyloric stenosis) and respective mice do not show evident histopathological abnormalities (27). These results suggest that inhibitory neurons are also co-regulated by other neurotransmitters to terminate the excitatory signal. Several transmitters/neuropeptides have been described to regulate inhibitory motor neurons including adenosine triphosphate (ATP) (28), vasoactive intestinal peptide (VIP) (29), pituitary adenylyl cyclase activating peptide (PACAP) and carbon monoxide (30) in addition to NO as neuromuscular transmitters (31).

In summary, excitatory and inhibitory motor neurons in the ENS control muscle contraction and relaxation in an autonomous manner (**Figure 1**). In this way, ingested food is squirted, mixed with digestive enzymes and eventually transported aborally. Based on gene profiles detected in single-cell RNA-sequencing studies, neurons can be associated to either excitatory or inhibitory motor neurons. However, the exact functional role of the subpopulations within excitatory or inhibitory motor neurons remains elusive.

In this context, putative intrinsic but also extrinsic neurons are equipped with mechanosensory ion channels. Different cell types have been described to use mechanosensitive ion channels to detect alterations in mechanical forces. In this way, neurons can adapt their functions dependent on mechanical perturbations. Piezo ion channels are important signaling cationic ion channels for mechanosensation and their role in the extrinsic nervous system and the enteric nervous system are emerging (32). Functionally in the peripheral nervous system, PIEZO ion channels have been linked to the sensation of nociceptive signals, proprioception and touch. An interesting finding from an immunologic perspective is that mechanical processes can also regulate immune cell activity (33). The authors found that the mechanosensory ion channel PIEZO1 is able to mount a proinflammatory reprogramming in macrophages. Upon conditional deletion of PIEZO1 in myeloid cells in the context of *P. aeruginosa* infection in the lung, the PIEZO1-mediated mechanosensation protected against bacterial infection (33). This data argues for similar mechanisms used by the nervous and the immune system to adapt physiological functions to mechanical forces. Thus, mechanosensory ion channels may be important for neuro-immune disturbances in ileus and other gastrointestinal diseases. However, their exact role, in particular the role of PIEZO1 in the gastrointestinal tract, needs further investigation.

### Secretomotor and Vasodilator Neurons

The intestine is constantly exposed to potential external threats, such as the commensal microbiota, in addition to the needs to control bowel absorption and to avoid electrolyte disturbances. Intrinsic secretomotor neurons have their cell bodies predominantly in the submucosa and play an important role in the regulation of these functions (**Figure 1**). In the guinea-pig small intestine, three different *ChAT* expressing secretomotor/vasodilator neurons have been described. Co-expression of *ChAT* and Calretinin, both of which innervate glands and arterioles, has been linked to vasomotor function. Functionally, excitation of vasomotor neurons lead to vasodilation and, thus, increase local blood flow (34). This is interesting because the topical application of Ach on vascular smooth muscle normally causes vasoconstriction and thus Ach seems to exert context-dependent effects. The two other neuronal classes described in the guinea-pig can be functionally assigned to different secretomotor neuronal populations. These neurons and their associated regulatory mechanisms are of great importance in host physiology because interference with secretory mechanisms may lead to constipation or diarrhea. Secretomotor neurons are activated *via* intrinsic primary afferent neurons following chemical or physical interactions with luminal contents (34). Such stimuli can activate cAMP or Ca^2+^ activated chloride ion channels, which regulate the movements of chloride towards the intestinal lumen or through epithelial cells into the lamina propria and cause water to diffuse along (35, 36). Based on expressed neuro-transmitters, two types of secretomotor neurons can be distinguished; the first group co-expresses *ChAT* and neuropeptide Y, whereas the second group is characterized by the expression of *ChAT* only (37). However, future studies need to delineate the functional role of each neuro-transmitter in detail, which will allow to correctly assign the transcriptional profiles to neuronal classes.

### Sensory Neurons

The ENS needs to monitor mechanical and chemical perturbations in order to react to incoming physiologic or pathologic signals and to tune local cellular components (**Figure 1**). Extrinsic sensory innervation includes spinal and vagal afferent neurons, whose cell bodies lie outside the intestine, e.g. in the Dorsal Root Ganglia or in the nodose/jugular ganglia. While anatomically defined, distinct afferent neurons originating from the intestine project into dorsal root ganglia, the vagus nerve innervates the entire intestine with a denser innervation in the small intestine as compared to the large intestine. Both types of extrinsic axons, DRG-derived or vagal, project into the inner and outer layers of the intestinal wall and perceive signals that are important to guide homeostatic functions. The respective neuronal population and its functional role are discussed in the next paragraph (Dorsal Root Ganglia section of the review).

Intrinsic innervation is characterized by cell bodies lying inside the gut wall. Neurons that perceive and integrate sensory information in the ENS are called intrinsic primary afferent neurons (IPANs). IPANs are integrated in the neuronal architecture to act in concert with motor neurons, interneurons and secretomotor neurons to direct homeostatic functions depending on external stimuli and the needs of digestive functions (38). Similar to the ENS, sensory neurons can be classified according to their neurochemical signature. Sensory IPANs have been identified based on the known markers *Calca*/*Calcb*/*Nfem*/*Calb1*/*Calb2* and the selective expression for *NMU*, *Ucn-3/Cck* or *Nxph2*.

Depending on the function of the neurons, three types of IPANs are identified using small intestine of the guinea pig as a model organism. First, chemosensitive IPANs respond to chemicals present on the surface of the small intestine (39). Because nerve fibers do not reach the surface of the intestine, and thus, do not come into direct contact with luminal contents, chemical changes in the intestinal lumen have to be sensed indirectly *via* signals from epithelial cells (40). Enteroendocrine cells are specialized epithelial cells, which for instance release 5-Hydroxytryptamine (5-HT) upon mucosal chemical or mechanical stimulation, which is a potent stimulator of IPANs and act as a signal transducer (41). However, knowledge concerning how endothelial cells communicate with the ENS remains a black box. Analysis of single-cell RNA-sequencing data from epithelial cells suggest the presence of potential stimulating peptides, such as Substance P, cholecystokinin, ghrelin, and synthesizing enzymes of signaling amines (5-HT) in these cells (42). In line with these observations, receptors for the respective stimulators, such as the 5-HT_3_ receptor and Substance P receptor 1, is expressed in IPANs suggesting an enteroendocrine to ENS signaling hub. Thus, sensory neurons functionally assigned to chemosensitive IPANs could be identified by a distinct gene profile for receptors of stimulating peptides and amines. However, the stimulation of other classes of IPANs, the mucosal mechanoreceptors, is also mostly indirect *via* 5-HT released from enterochromaffine cells (43). The second functional class described are stretch-sensitive IPANs that react to mechanical tension/distortion (38). Interestingly, these neurons seem to be not only mechanosensitive, but can also directly act as inhibitory motor neurons (44). Thus, their gene profile may be mixed and complicated for a functional assignment. The third group of IPANs are mucosal mechanoreceptors, which may be identified by putative mechanosensitive ion channels (e.g. Piezo2). However, as stated above, the stimulation of mucosal mechanoreceptors is mostly indirect and further studies need to unravel their profiles in more detail.

### Interneurons

The interneurons, as the name suggests, are neurons, which connect functionally diverse neuronal populations in order to complete a neuronal circuit of varying complexities. For instance, interneurons receive signal from sensory neurons (which sense environmental perturbations) and relay the signal to either inhibitory or excitatory motor neurons to trigger an effector function to the sensed stimulus (**Figure 1**). The interneurons, as a type of 'bridging neurons', are multipolar and can be excitatory or inhibitory. They are primarily located within the myenteric plexus forming uniaxonal chains along the length of the gut with the ascending interneurons projecting orally and the descending interneurons projecting anally. There are different interneurons within the myenteric plexus with distinct neurochemical signatures, which can differ between gut regions. Using the guinea pig as a model organism, one class of excitatory ascending interneurons and three classes of descending interneurons have been described in the small intestine (45). In the colon on the other hand, three neurochemical classes of ascending interneurons and four classes of descending interneurons have been identified (46). These anatomic differences may come from the distinct local environment (e.g. microbiota) and the functional role it has to execute in different anatomic regions. The method applied by the authors was to morphologically distinguish interneuronal subpopulations. Whereas, upon distinguishing the subpopulations of interneurons based on their transcriptional signature, Morarach et al. was able to identify two subpopulations: one expressing motor-neuron-like inhibitory and excitatory neuropeptides *Nos1*/*ChAT* and the other expressing sensory-neuron-like neuropeptides *Sst*/*Calcb*/*Calb2* (13). The discrepancy in the findings/characterization of these studies might be a result of difference in either their method of evaluation (morphological vs transcriptomic) or the anatomic locations within the gut studied. However, several studies demonstrate that neurochemical markers of the interneurons overlap with other neuronal populations and, as such, do not have their unique neurochemical signature.

## Integration of Sensory Neuronal Signals Originating From the Luminal Content of the Intestine

Since nerve fibers do not reach the lumen of the intestine under homeostatic conditions, microbiota and metabolites could directly stimulate neurons by penetrating the epithelial barrier or indirectly through epithelial cells or other cell types. Epithelial enteroendocrine cells were shown to be directly innervated by neurons and transduce signals to the CNS within milliseconds after being exposed to sugar (47). Although the finding that epithelial cells are innervated is still controversial (48), sensing of secondary signals released from epithelial cells by enteric neurons is likely to contribute to the regulation of intestinal homeostasis.

Using AAV particles for neuronal-specific deletion of aryl hydrocarbon receptor (Ahr) in enteric neurons, Obata et al. could demonstrate a pivotal functional for enteric neurons in metabolite sensing (49). Genetic ablation of Ahr resulted in reduced peristalsis and increased intestinal transit times. Further, Ahr expression was altered in germ-free mice suggesting a role for commensal microbiota in regulating Ahr expression in neurons. Several studies have reported that the absence of an intact microbiota resulted in activation of neurons and alterations of neuronal composition in ENS (48–50) as well as effects on CNS function were reported (51, 52). This leads to the question if and how neurons sense microorganisms. In vitro data show that neurons are able to respond to PAMPs, for example LPS *via* an TLR4-independent, or excretory secretory product of helminth in an Myd88-dependent manner (53, 54). Analysis of enteric neuronal populations in germ-free mice has revealed alterations in the enteric nervous system, in particular of NOS1^+^ neurons, although there is some discrepancy between the studies whether NOS1^+^ neurons are over- or underrepresented (50, 55, 56). Similar findings were reported in Myd88-deficient mice arguing for a role of TLRs in microbial sensing and development of the ENS. However, it remains unclear if the phenotype is explained by direct sensing of neurons *via* TLRs or indirect mechanisms *via* secondary messengers. Conditional gene targeting of TLR4 using WNT1-Cre resulted in altered ENS development. However, it should be mentioned that this targeting strategy does not exclude a major role for glial cells in TLR4 microbial sensing instead of or in addition to enteric neurons (13, 50).

Investigating neuronal-specific gene expression in germ-free animals by using the Ribotag system activated by SNP25Cre, Muller et al. could detect cell death of CART^+^ neurons, which are involved in regulation of blood glucose levels, mediated by NLRP6 and Casape-11-dependent pathway (20). Further, the absence of an intact commensal microbiota is detected in parts *via* SCFA and GPR41 resulted in an increased sympathetic neuronal activity, as measured by c-FOS expression in the coeliac-superior mesenteric ganglia, activation of glutamatergic sensory neurons in the brainstem and decreased intestinal transit time (48). Similarly, cell death controlled by a NLRP6 and Casape-11-dependent pathway as well as sympathetic activation have been reported following *Salmonella enterica* infection resulting in decreased intestinal motility. In this context, activation of the sympathetic nervous system and release of norepinephrine can instruct a tissue-protective program in muscularis macrophages characterized by Arginase 1 and BMP-2 expression and responsiveness to enteric neuron-derived CSF-1 (48, 57).

Altogether, the signaling circuits sensing mucosal homeostasis are emerging and the integration of these signals in the CNS can provide on more holistic view on how intestinal homeostasis is regulated.

## Dorsal Root Ganglia

**Figure 5:**
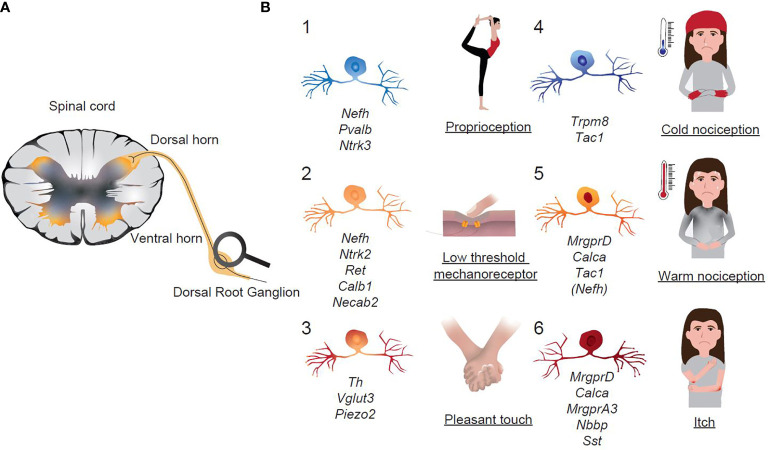
Functional classification of sensory neurons in Dorsal Root Ganglia. **(A)** Schematic representation of extrinsic sensory afferent neurons that transmit signals from the periphery to the CNS. **(B)** Schematic representation of sensory neuronal groups in DRGs with assigned functions. Italics are the key transcripts of the respective neuronal group.

Dorsal root ganglia (DRGs) comprise of neuronal cell bodies of sensory neurons, whose nerve fibers innervate different anatomic regions (e.g. thorax, small intestine, colon and extremities). The cell bodies represent the somas of the first-order sensory neurons and constitute an integral part of the somatosensory system. DRGs process sensory information transduced from afferent nerves, which may include nociceptive information, in addition to signals generated in steady-state conditions such as the feeling of discomfort and satiety (**Figure 5**). The afferent neurons of DRGs integrate sensory information from distinct body regions and transmit the signal to the CNS (58). Sensory afferent signals from the thoracolumbar region predominantly originate from the small intestine and lumbosacral DRGs mainly project the lower extremities, but also to the large intestine (59–61). Different types of sensory neurons within DRGs allow sensing of distinct stimuli. Initially, sensory neurons were categorized based on their degree of myelination and the associated conduction velocity. This classification led to four main classes of neurons, namely thinly myelinated Aδ fibers, unmyelinated C fibers, heavily and moderately A myelinated fibers. Because of a significant heterogeneity in the degree of myelination and conduction velocity in functionally similar classes, this classification insufficiently reflects function. To gain further insight into different populations and to functionally discern the neurons within DRGs, there are now many studies available, which have performed single-cell RNA-sequencing and associated gene expression with a function in mice, primates and humans (12, 62–68). The respective datasets can be accessed *via* online tools and screened for genes of interest (as outlined in **Table 2**).

**Table 2:** links to access the respective datasets.

Study	Sequencing site	Links
Zeisel et al. (12)	ENS	http://loom.linnarssonlab.org/dataset/cellmetadata/Mousebrain.org.level6/L6_Enteric_neurons.loom
Usoskin et al. (63)	DRG naive	http://linnarssonlab.org/drg/
Sharma et al. (68)	DRG naive	https://kleintools.hms.harvard.edu/tools/springViewer_1_6_dev.html?datasets/Sharma2019/all
Zeisel et al. (12)	DRG naive	http://loom.linnarssonlab.org/dataset/cellmetadata/Mousebrain.org.level6/L6_Peripheral_sensory_neurons.loom
Renthal et al. (69)	DRG after axonal injury	http://www.painseq.com
Hockley et al. (70)	DRG colon	http://hockley.shinyapps.io/ColonicRNAseq.
Häring et al. (71)	Dorsal horn of spinal cord	https://linnarssonlab.org/dorsalhorn/

Single-cell RNA-sequencing, if not used with any selection, is an untargeted and unbiased approach that allows identification of neuronal cell types based on transcripts expressed by the cells. However, there is a certain noise in between studies that complicates the interpretation of the results. Furthermore, a clear limitation of current techniques is the rather superficial sequencing depth at a single-cell level and the required dissociation of cells, which may omit large and long axonal neurons during sort-purification. In future studies, such limitation may be overcome by the single-nuclei sequencing approach as described above (14).

However, with the available data, neurons can be classified into neurofilamentous, peptidergic and non-peptidergic neurons, which are uniformly detected in all the above-mentioned studies. A recent study used a machine-learning approach for three available nomenclatures/classification of DRG neurons (12, 63, 68) in finding the corresponding cell types in different datasets (**Table 3**) (64). The Usoskin classification showed the least 'noise' and highest prediction score when comparing different datasets and may therefore be the classification of choice (64). However, it has to be mentioned that the Usoskin study detected fewer neurons (622 neurons) compared to the work of Zeisel (1'580 neurons) or Sharma (10´922 neurons). The Usoskin-classification proposed a classification of DRG neurons into a total of 11 groups: 5 subgroups within the neurofilamentous group (NF1-5), 4 non-peptidergic neurons including TH^+^ neurons (NP1-3, TH), 2 peptidergic neurons (PEP) including Nav1.8^+^ neurons (**Table 3**) (63). Another study by Zeisel et al. was consistent with the results obtained by Usoskin, but due to more sequenced neurons, sub-clusters within peptidergic neurons (PEP1, 2, Trpm8), non-peptidergic neurons (NP 1, 2) were identified (12). One has to consider that available single-cell RNA-sequencing studies may compare DRGs that receive afferents from different anatomic locations and the subtype composition may vary across axial levels, which therefore could explain minor discrepancies when comparing neuronal population datasets (68, 70). Thus, sequencing of neurons isolated from either thoracic, lumbar or sacral regions may unravel anatomic fingerprints of gene expression in future studies.

**Table 3:** Reference genes for the identification of neuronal subsets in DRGs based on unbiased single-cell RNA sequencing (adapted from (12, 58, 63, 64, 68).

	Proprioceptive/touch sensation neurons				Nociceptive neurons														
					Non-peptidergic						Peptidergic neurons								
Usoskin	NF1	NF2	NF3	NF4	TH	NP1	NP1	NP2	NP2	NP3	Pep2	Pep2	Pep1	Pep1	Pep1	Pep1	TRPM8	TRPM8	TRPM8
Zeisel	NF1	NF2	NF2	NF3	NP1	NP2	NP3	NP4	NP5	NP6	Pep1	Pep1	Pep2	Pep3	Pep4	Pep5	Pep6	Pep7	Pep8
Sharma	Adelta-LTMR	Abeta-RA-LTMR	Abeta-Field	Proprioceptors	C-LTMR	NP- nociceptors	NP-nociceptors	CGRP-theta	CGRP-theta	Sst	CGRP-zeta	CGRP-ota	CGRP-gamma	CGRP-epsilon	CGRP-alpha	CGRP-beta	TRPM8	TRPM8	TRPM8
Signature genes	Nefh	Nefh	Nefh	Nefh	TH	Mrgprd	Mrgprd	Calca	Calca	Tac1	Calca	Calca	Calca	Calca	Calca	Calca	Trpm8	Trpm8	Trpm8
	Ntrk2	Ntrk2low	Ntrk3high	Pvalb	Vglut3	Prkcq	Prkcq	MgprA3	MgprA3	Nppb	Nefh	Nefh	Tac1	Tac1	Tac1	Tac1	Tac1	Tac1	Tac1
	Necab2	Ret	Fam19a1	Ntrk3	Piezo2	Agtr1a	Agtr1a	Gfra1	Gfra1	Nts	Ntrk1	Ntrk1	Sertm1	Ltk	Sstr2	Dcn	Angpt4	Ntm	Pnoc
	Cacna1h	Calb1	Ret	Runx3	Zfp521	Lpar3	Cyp26b1	Mlc1	Mlc1	Il31ra	Smr2	Smr2	Mrap2	Traf3fp3	Dcdc2a				Penk
						Barx2				Osmr	Creg2	Creg2	Slc5a7						

NF 1-4, neurofilamentous neurons; TH, thyroxin hydroxylase; NP1-6, non-peptidergic neurons; Pep, peptidergic neurons; TRPM8, transient receptor potential cation channel subfamily M member 8 neurons; LTMR, low threshold mechanoreceptor.

Another recent study identified a total of 12 classes of DRG neurons, which consists of Aβ-rapidly adapting (RA) low threshold mechanoreceptors (LTMR), Aδ-LTMR, C-LTMR, 6 groups of Calcitonin Gene-related Peptide (CGRP) neurons, Mrgrd^+^ polymodal nociceptors, Somatostatin^+^ and cold thermosensors (68). Interestingly, a direct comparison of the three main classifications (Usoskin et al. *vs* Zeisel et al. vs Sharma et al.) by the before mentioned machine-learning approach found a high probability of finding a distinct cell across different datasets (64). Thus, the same cell can be found in different datasets and the difference between studies relies on the annotation and is not based on biologic differences. All the above-mentioned DRG-related studies have been performed in naïve animals and no specific disease model was profiled in detail. Therefore, it is of great interest to decipher transcriptional regulators in disease states such as nerve injury and concomitant neuronal regeneration at a single-cell resolution. A recent single-cell sequencing paper describes interesting features associated with different nerve injury models (69). First, a reduced expression of neuronal-subtype marker genes, such as *Tac1*, *Mrgprd*, and *Nefh* is described. Second, but not unexpected, genes involved in axon guidance, axogenesis and cell migration have overall increased. Of particular interest are specific transcripts found in nociceptive neurons because of their role in the development of neuropathic pain (69). Genes of interest can be browsed in the available online tool (see **Table 2**).

A large set of publications has studied different neuronal molecules, which help to propose a relation of the molecular profile of the respective neuron with modality-specific function within DRG neurons. These studies allow to functionally predict the different neuronal populations found in single-cell RNA-sequencing studies (**Figure 5**). Generally, neurofilamentous and TH populations are proprioceptors (control of body positioning and balance) and LTMRs (touch sensations), whereas non-peptidergic and peptidergic neurons are nociceptors (damage/potential damage signals), which represent the majority of all DRG neurons. Within the group of nociceptors and based on the molecular signature, a broad functional sub-classification is made: TRP cation channel subfamily member 8 (TRPM8) neurons are cold-sensory neurons, peptidergic neurons detect heat and pain, isolectin4-binding non-pepdidergic neurons can sense noxious touch, itch and chemical signals (**Figure 5**).

Because of available top gene transcripts, the populations can be identified and targeted, either *via* immunohistochemistry, potentially flow cytometry or used for a conditional deletion by using specific Cre-drivers in mice that allow deleting genes within a neuronal subpopulation.

### Colonic Afferent DRGs

A recent single-cell RNA-sequencing study on retrogradely traced colonic sensory neurons in the mouse identified seven neuronal subtypes (70). The authors identified 5 specific subtypes in the thoracolumbar region and seven subtypes in the lumbosacral region, two of them being exclusively found in the lumbosacral region. The populations identified in both anatomic regions included Neurofilament-a and Neurofilament-b, which express genes typically associated with myelinated DRG neurons, such as neurofilament heavy chain (*Nefh*) and lactate dehydrogenase B (*Ldhb*). The third subtype was classified as non-peptidergic neurons and showed an expression pattern of non-peptidergic nociceptors, such as the purinergic receptors P2X3 or glial-cell line derived neurotrophic factor family receptor alpha2 (*Gfrα2*). The last two subtypes express *Calca*, *Tac1* and *TrkA* and have been termed as peptidergic nociceptors. The subtypes of neurons exclusively found in the lumbosacral region included a neurofilament and a peptidergic subtype, both of which show a relatively similar basic expression pattern (neurofilament subgroup: *Piezo2*, *Nefh*, *Ldhb*; peptidergic subgroup: *Calca*, *Tac1*) but are furthermore characterized by a specific gene set suggesting that neurons have an anatomic fingerprint.

### DRGs Originating From Iliac/Skin-Innervating Lymph Nodes

Apart from sensory neurons directly originating from organs such as the intestine, lymph nodes show a spatial distribution of sensory and sympathetic neurons (72). A more comprehensive view by using tracing experiments *via* the injection of Cre-expressing viruses into iliac lymph nodes and single-cell RNA-sequencing of labelled DRG neurons reveals 4 transcriptionally distinct neuronal subtypes (72). The vast majority of detected neurons express *Nav1.8* and only few co-expressed *TH.* By comparing the detected neurons to publicly available datasets (63, 68), the study revealed that lymph nodes are equipped with transcriptionally heterogenous, predominant peptidergic nociceptors. Of great interest in inguinal-lymph nodes are particularly enriched genes involved in inflammation and neuro-immune-communication, such as *Il33*, *TLR*s or *Ptgir*. The same authors also observed sensory neuronal remodeling after LPS application suggesting modular sensing of danger signals by the nervous system (72). Future studies should focus on the sensory DRGs of mesenteric lymph nodes to delineate transcriptional profiles associated with the primary hubs exposed to constant PRR signals.

### Vagal Ganglia

Apart from DRGs located in close proximity to the spinal cord, vagal ganglia receive afferent signal input and mediate host protection against a broad variety of sensations. The upper airways are densely innervated by the vagus nerve with the fused primary order neurons located in the nodose/jugular/petrosal superganglia clustering near the jugular foramen. These sensory neurons play a pivotal role in swallowing reflex pathways and coordinate the upper airways to prevent aspiration pneumonia or dysphagia (73). Single-cell RNA-sequencing of nodose/jugular/petrosal ganglia with a coverage of 25'117 sensory neurons (spanning 2293 genes) revealed a total of 37 classes of neurons. *P2RY1^+^* neurons seem to be particularly relevant with respect to swallow reflexes because conditional ablation of P2RY1 neurons led to impaired swallowing responses (73). Given the wide variety of neurons located in vagal ganglia, the role of other subclasses remains elusive and needs to be studied in future projects.

## Functional Role of Neuronal Subpopulation in DRGs

### Neurofilaments-Expressing Neurons (Groups NF1-4)

Neurofilaments (NF, gene *Nefh*) are 10nm bundles of fibrils within neurons (74). In contrast to the perikarya, NF can be highly abundant in axons (75). In concert with microtubule and microfilaments, NF form the neuronal cytoskeleton and are aligned in parallel along the axonal axis. The filaments primarily support neuronal structure and regulate the axonal diameter, which is a critical determinant of neuronal conduction velocity (76, 77). Based on the marker NF200, two distinct neuronal subtypes have been identified that allow the discrimination between large myelinated or thinly myelinated neurons with fast conduction velocities (NF200 positive) and small unmyelinated neurons with low conduction velocities (NF200 negative) (78). Because of their high conduction velocities, NF neurons are involved in signals that require fast signal transmission, such as touch sensations and proprioception. Apart from NF, spinal proprioceptive neurons, which innervate muscle spindles and Golgi tendon organs, are further characterized by the expression of neurotrophin-3 (*Ntf3)* and parvalbumin (*Pvalb*) (79). The expression of other marker genes, such as Tropomyosin receptor kinase A and B (*TrkA*, *TrkB*), Ret, calbindin (*Calb*), functional predictions can be made of NF neurons and allow for a certain functional classification (58).

According to the Usoskin-classification, gene expression profiles of NF group 1-3 is characterized by a pattern of *Nefh*, *Ntrk2*, *Ret*, *Calb1* and *Ntrk3* (63). These groups can be functionally assigned to low threshold mechanoreceptors (LTMRs). The expression of *Ntrk3* and *Pvalb* in NF groups 4/5 suggests that these neurons have a proprioceptive role (**Table 3**) (63). In general, proprioceptors are located within the musculoskeletal system and relay the information to the CNS concerning body position and movements (**Figure 5**).

A more comprehensive view into the subgroups and the associated gene expression suggests that NF group 1 are lightly myelinated Aδ LTMRs and very sensitive velocities detectors tuned to the deflection of body hairs (80). NF group 2 are rapid adapting LTMRs that end in Meissner corpuscle and longitudinal lanceolate endings and are critical for the perception of skin movement and vibration (81). Because of the high expression of *Ntrk3*, NF group 3 can be assigned to slowly adapting LTMRs, which control the perception of stretch and indentation (81).

### TH Neurons

TH-expressing DRG neurons have been shown to be C-LTMRs (82). One of the top genes expressed in this population, *Vglut3*, has been identified to be uniquely found in C-LTMRs. Functionally, absence of Vglut3 in *Vglut3* ^-/-^ mice has been linked to defects in acute mechanical pain sensations upon intense noxious stimuli (83). An additional feature of these neurons is the high expression of the mechanosensitive ion channel, *Piezo2*. Apart of mechanical pain, C-LTMRs perceive low mechanical forces such as pleasant touch of the skin.

### Peptidergic and Non-Peptidergic Neurons

Slow conducting DRG neurons are classified into peptidergic and non-peptidergic neurons. Peptidergic neurons are defined based on the expression of the neuropeptides substance P, calcitonin gene-related peptide (CGRP), and somatostatin, whereas non-peptidergic neurons bind the plant lectin IB4, express the Mrg family of G-protein coupled receptors and P2X3 (84, 85). However, one must take into account that all of these markers are only partially selective and some overlap can exist (86). Basically, peptidergic and non-peptidergic neurons are unmyelinated, primary afferent neurons assigned to nociceptors and thus respond to damage and potential damage stimuli (**Figure 5**).

From an immunologic perspective, peptidergic neurons and their secreted neuropeptides, including Substance P, CGRP and NMU, have a marked immunoregulatory potential, which has been shown by many studies (54, 87–91) and will be discussed in detail in a later paragraph.

#### Non-Peptidergic Neurons

The expression of the Mas-related G-protein coupled receptor member D (*MrgprD*) in non-peptidergic neurons group 1 (NP1) suggests its role in the perception of noxious mechanical and thermal stimuli as well as in the perception of itch (92, 93). NP2, expressing *MrgprA3* and *Calca*, are polymodal nociceptors and evoke itch responses (94). Both groups, NP1 and NP2, have been linked to the development of neuropathic pain (63, 95, 96). Even though characterized by a different set of key genes, NP3 have similar functions than NP2 and are also itch-sensing neurons. Relevant genes in this neuronal subset include the natriuretic polypeptide B (*Nbbp*) and Somatostatin (*Sst*). Injection of Nbbp intrathecal triggered itch responses in mice whereas itch-responses were blocked upon ablation of Nbbp-receptor-expressing cells (97). In the same line, Sst co-expressed with Nbbp neurons and Sst^+^ neurons triggered itch behavior (98).

#### Peptidergic Neurons

Pep1 neurons can be largely grouped by the expression of *Tac1*, which represents the gene for substance P. In terms of neuronal function, this neuronal population is involved in thermosensation and thus reacts to noxious heat and cold stimuli (63, 99, 100). However, substance P has also a major role in neuro-immune interactions, which will be discussed in a later paragraph. Similarly, these neurons express CGRP (*Calca*), which has also been implicated in regulating immune functions. Pep2 neurons express neurofilament heavy chain (*Nefh*) as well as *Ntrk1*, suggesting that these neurons are lightly myelinated Aδ nociceptors, which have a higher conduction velocity than unmyelinated neurons (63). Thus, these neurons respond to dangerously intense mechanical or mechanothermal stimuli. TRPM8 neurons are involved in thermal perception of temperatures <20°C and cold-triggered nociception (101).

## The Dorsal Horn of the Spinal Cord

Somatosensory sensations, as described above, can be activated by a large variety of stimuli. Excitatory and inhibitory afferent neurons transmit the signal to DRGs, where a first processing of the signal input occurs. Afferents from DRGs relay the information to the CNS *via* interneurons located in the dorsal horn of the spinal cord. The incoming information is further processed within the dorsal spinal cord and ultimately relayed to different brain areas (102). A recent study using single-cell RNA-sequencing of the dorsal spinal cord identified that all neurons either express the vesicular glutamate receptor 2 (Vglut2) or the vesicular GABA transporter (Vgat), thus, representing glutamatergic excitatory or GABAergic inhibitory neurons. To enrich neurons, the authors used the reporter mouse lines Vgat^tdTom^ and Vglut2^tdTom^ and FACS-enriched the samples for the respective neuronal subsets (71). The final dataset of 1'545 neurons revealed 15 glutamatergic and 15 GABAergic neuronal subsets and based on literature, the expressed transcripts have been linked to certain physiologic functions (71). For detailed information, we kindly refer to the original articles since the complexity of spinal cord neuronal population is beyond the scope of this review (71, 103). A very interesting novel feature that allows to monitor activation of cell types involved in sensory signal transmission is the sensory-transcription coupling. By using triple in-situ hybridization and the combination of the immediate-early gene expression of *Arc* with markers for distinct neuronal cell types revealed that noxious heat and cold activate different sets of excitatory and inhibitory neurons (71). This finding underlines the complexity of neuronal networks and argues for a broad activation of neurons in response to one distinct modality.

## Neuro-Immune Signaling in Host Physiology and Disease

Enteric and DRG neurons express the neuro-peptides VIP, CGRP, Substance P, and NMU, and the neuro-transmitters norepinephrine and acetylcholine. Many studies identified the respective receptor expression on a large set of immune cells, which implies the regulation of immune responses by the nervous system. Furthermore, the close co-localization of hematopoietic cells with neuronal fibers suggests a bidirectional signaling exchange. These neuro-immune modules constantly interact in order to adapt physiologic processes. The following paragraph reviews neuro-immune interactions based on neuro-peptides or transmitters.

### Vasoactive Intestinal Peptide (Gene: *VIP*)

VIP engages on two subsets of receptors, Vipr1 and Vipr2. Thus, solely studying the experimental effects of VIP may be difficult to interpret because of receptor-dependent responses. Furthermore, mechanisms behind such studies do not allow to conclude specific effects on a cellular level. This fact has been observed in the following experimental designs: On one hand and in the context of gastrointestinal inflammation, VIP knock-out mice showed a more severe phenotype in DNBS- and DSS-induced colitis in mice and VIP has been linked to the maintenance of intestinal integrity (104). On the other hand, models of DSS-induced colitis in *Vipr1* ^-/-^ and *Vipr2* ^-/-^ mice displayed opposite results. In fact, *Vipr1* ^-/-^ mice showed a milder disease score compared to wild type mice, whereas *Vipr2* ^-/-^ developed a more severe colitis (105, 106). Thus, studies addressing the role of VIP need to take into account the distinct affinity of VIP onto its receptors and the distinct receptor expression on a broad array of immune cells. *Vipr2* is expressed by a large set of immune cells including innate lymphoid cells type 3 (ILC3), which play a crucial role in host defense mechanism against bacteria. Recent studies have now described *Vipr2* expression by ILC3s and binding of the ligand VIP regulates the release of IL-22 (106, 107). Because IL-22 controls the antimicrobial peptide production and regulates lipid absorption, the VIP-IL-22 axis has been proposed to shift the balance in host defense mechanisms and lipid uptake (107). Furthermore, VIP and IL-22 is released in a feeding-dependent manner and, thus, underlines the role of neuro-immune signaling for adaptations of barrier mechanisms dependent on food-uptake. However, the role played by VIP on ILC3s seems to be context dependent acknowledged by controversial results published by different authors (106, 107). VIP has also been shown to regulate type 2 immune reactions in the lung (108). By using ovalbumin-induced lung inflammation, the authors showed an increase of VIP transcripts in nodose ganglia of inflamed lungs, which was dependent on nociceptive neuronal firing. Upon release, VIP engages to Vipr2 and activated the release of type 2 cytokines from ILC2 and CD4^+^ T cells (108). In the same context, nociceptors express FcϵR1 and directly sense IgE-OVA complexes to initiate type 2 immune reactions (109). In summary, VIP appears as an important regulator of type 2 and type 3 immune responses.

### Calcitonin Gene-Related Peptide (Gene: *Calca* and *Calcb*)

By using Capsaicin-induced denervation in neonatal mice and immunoassays, early studies already suggested that the vast majority of CGRP neurons have an extrinsic source in the intestine (110). According to current knowledge, the main source of CGRP are the unmyelinated sensory C fibers in DRGs (111). As reviewed above, CGRP-expressing neurons are assigned to nociceptive neurons and, thus, sense damage and potential damage stimuli and are involved in the perception of pain. Because CGRP expressing nerve fibers have been found in many immunologic organs including bone marrow, spleen, lymph nodes, skin and the intestine, and the receptor is expressed on many different immune cells, the immunoregulatory potential of CGRP has been repeatedly shown (54, 88, 89, 111–113). Following the release, CGRP engages the calcitonin receptor-like receptor (CALCRL) and the receptor-modifying protein 1 (RAMP1). It is important to note that immune cells have also been shown to express CGRP, which suggests a bi-directional crosstalk between immune cells and the nervous system (114, 115). In general, CGRP seems to have anti-inflammatory effects in the GI-tract. This has been highlighted in gain-of function experiments by modelling inflammatory bowel disease and the systemic administration of CGRP in rats, which resulted in an amelioration of TNBS-induced colitis (116, 117). In line with these results, loss-of-function experiments by using CGRP antagonists in rats or CGRP knock-out mice revealed increased susceptibility to colitis (118, 119). These results suggest that CGRP agonists may be potential therapeutic targets for the treatment of inflammatory diseases. The anti-inflammatory role of CGRP has also been highlighted in the context of *Streptococcus pyogenes* infection in the skin, which is the leading cause of the life-threatening necrotizing fasciitis. The authors found that S. pyogenes directly secretes streptolysin S and promotes the release of CGRP from nociceptors, which inhibits neutrophil recruitment and phagocytic killing (120). In the same line and in the context of *Salmonella enterica* infection, TRPV1^+^ nociceptors release CGRP and modulate M cells to eventually control host defense against Salmonella (121). In terms of type 2 immune reactions, innate lymphoid cells type 2 (ILC2) express the receptors CALCRL/Ramp1 and binding of CGRP modulate ILC2 activation, whereas deletion of this signaling cascade elevated ILC2 responsiveness and type 2 immunity (88, 89, 113). Thus, the prevailing view regarding the role of the neuropeptide CGRP is its overall anti-inflammatory properties. With regard to many overwhelming immune reactions, topic or systemic application of CGRP may be a valuable treatment strategy. However, further studies need to investigate the related mechanisms in more detail.

### Substance P (Gene: *Tac1*)

Substance P (SP) is a member of the tachykinin family of neuropeptides and is encoded by the Tachykinin 1 (*Tac1*) gene. SP exerts its function *via* the engagement on G-protein coupled neurokinin receptors but predominantly binds on the neurokinin 1 receptor (NK1R) (122). SP is expressed in the central and peripheral nervous system and intrinsic neurons seem to be the major source in the intestine (123, 124). Once synthesized, SP is transported in large dense-core vesicle and released *via* exocytosis where it exerts its function on the same cell or the adjacent cell (125). Apart from neurons, immune cells have been implicated to express both, SP and its receptor NK1R (126). The relative broad expression of the receptor on immune cells in the lamina propria, such as mast cells, eosinophils, neutrophils, macrophages, dendritic cells and natural killer cells implies its tight regulatory immune function. In general, SP has a pro-inflammatory role *via* the induction of pro-inflammatory cytokines in immune cells (127). In particular, it has been highlighted that SP and its receptor NK1R increase the susceptibility to DSS- and TNBS-induced colitis (128, 129). A newly developed intestinal organ culture system observed an anti-correlation of Tac1 and its receptor TacR1 after the application of different microbiota strains into the in vitro system. This negative correlation has then been linked to alterations of Rorgt^+^Tregs. Such observations suggest the potential of microbiota-sensing by Tac1^+^ neurons and the consecutive modulation of immunologic reactions (130). In the allergic setting, nociceptors release SP after allergen exposure and promote migration and activation of adaptive type 2 immune responses (131). However, because of its broad expression in several tissues, the exact cellular role of SP/NK1R has to be further studied in conditional deletion models in order to decipher the mechanism behind the observed phenotypes.

### Neuromedin U (Gene: *NMU*)

NMU is a short neuropeptide with highly conserved amidated C-terminus required for receptor binding. Neurons of the CNS, the pituitary gland and the ENS express NMU (132). Within the ENS, NMU labels together with CGRP a subset of ChAT^+^ sensory neurons (12, 53, 132). Further, NMU expression is regulated by the commensal microbiota and modulated by secretory excretory products of helminths (48, 53, 133). To mediate its biological function, NMU binds to two large G-protein coupled receptors Nmur1 and Nmur2. Nmur2 is expressed in neurons in particular in the CNS and regulates feeding-behavior, circadian rhythm as well as pain perception and bone formation (132). The immunomodulatory functions of NMU were recognized years ago (134–136), however, before ILCs were emerging. Thus, the mechanism remained elusive until several publications demonstrated that NMU acts specifically *via* ILC2s in different organs (53, 133, 137). By binding to Nmur1, NMU triggers a signal cascade *via* Gαq - PLC, activation of NFAT, resulting in activation of ILC2, proliferation and cytokine production. Upon NMU stimulation, ILC2 promote the type 2 immune response characterized by eosinophil recruitment, goblet cell hyperplasia and mucus production, resulting in increased worm expulsion during *N. brasiliensis* infection or enhanced airway inflammation following papain challenge. Altogether, these data indicate that sensory neurons regulate ILC2 activation *via* NMU and CGRP with downstream effects on various immune cell types participating in type 2 inflammation at mucosal barriers.

### Norepinephrine (Synthesizing Gene: Thyroxine Hydroxylase)

Norepinephrine has a dual function in mammalian hosts. On one hand, it is a stress or danger hormone, which is released during a fight-or-flight reaction and results in a concomitant increase in blood pressure, heart rate, glucose mobilization and other stress reactions. On the other hand, norepinephrine acts as a neurotransmitter released from sympathetic nerves located in sympathetic ganglia. Once released, norepinephrine binds and activates α- and β-adrenergic receptors, which are G protein-coupled receptors and thus exert their effect *via* a second messenger system. In general, β-adrenergic receptors seem to be immunologically more important given their anti-inflammatory effects in neutrophils, macrophages and ILCs (138–140). More specifically, β-adrenergic stimulation mediates the polarization of intestinal macrophages, which reside in close proximity to sympathetic neurons (138). After the activation of the β2-receptor, macrophages upregulate tissue protective-programs (138). Reciprocally, macrophages upregulate neuro-protective programs through an arginase1-polyamine axis, and, thus limit neuronal damage (57).

β2-adrenergic receptors are expressed in ILC2s and argue for a regulatory role of the sympathetic nervous system in controlling type 2 immune reactions. Indeed, norepinephrine has been shown to inhibit ILC2s, whereas ILC2 specific ablation of the β2 receptor magnified type 2 immune reactions and improved worm clearance in the context of *N. brasiliensis* infection (139). In summary, sympathetic neurons have an anti-inflammatory role in a broad range of immune cells.

### Acetylcholine (Synthesizing Gene: Choline Acetyltransferase)

Acetylcholine (Ach) is a neurotransmitter predominantly used in the autonomous nervous system and the major neurotransmitter of the parasympathetic nervous system. In addition, excitatory motor neurons, sensory neurons and interneurons within the ENS are capable of producing Ach. Ach mediates biological effects by binding to two different families of cholinergic receptors, the nicotinic and the muscarinic Ach receptors. Among immune cells, the nicotinic α7-Ach receptor is expressed on macrophages and ILC subsets and was investigated in detail due to its anti-inflammatory effects in various disease settings including but not limited to sepsis, IBD and arthritis. This intriguing finding, termed ‘cholinergic anti-inflammatory pathway’, uncovered a vagal regulated release of Ach, which binds on the nicotinic α7-Ach receptor on macrophages to suppress release of pro-inflammatory cytokines and in particular TNF-α, which is mediating immunopathology during sepsis or intestinal inflammation (141, 142). The cholinergic anti-inflammatory pathway has profound and rapid anti-inflammatory properties. The potent effects are illustrated by the prevention of septic shock in mice mediated by this pathway (143). Furthermore, stimulation of the vagal nerves using a medical device was successfully used in patients suffering from chronic arthritis to mitigate disease symptoms, which have been proven resistant to glucocorticoid treatment (144).

α7-nicotinic Ach receptor agonists were also shown to suppress ILC2 activation during allergic asthma (145). These data suggest a broad role of α7-Ach receptor signaling in damping chronic inflammation in various settings. On the contrary, vagotomy was shown to delay the resolution of inflammation following peritoneal *E. coli* infection. In this disease model, vagal stimulation promoted biosynthesis of resolvins in ILC3s (146). In addition, ILC3 might also serve as a source of Ach as described during allergic airway inflammation (147). In summary, Ach emerges as an important checkpoint for immune activation but the multifaceted actions as mucosal barriers require further investigation.

## Summary and Future Perspective

The identification of different neuronal subpopulations within the intrinsic ENS, as well as the extrinsic sensory compartment, provides the basis for studying the peripheral nervous system in detail. We noticed that certain genes are more ubiquitously expressed by different neuronal subpopulations and may therefore be involved in different functions depending on the neuronal subtype. However, our review combines several datasets and allows for the identification of neuronal subtypes based on genes expressed, morphology and function. The identification of key transcripts of neuronal populations now allows to design specific Cre-driver mouse lines to study neuronal programs in health and disease. There are three technical strategies available to study gene expression in neuronal populations: The desired Cre-driver mouse line can be crossed to a mouse with conditional expression of a fluorescent protein. Fluorescent cells can then be FACS-sorted und analyzed for gene expression (12). The other option is the nuclear dissociation and FACS-enrichment of neuronal nuclei as described recently (14). And the third option is the use of Ribotag mice, which have a hemagglutinin-tagged ribosomal protein specifically active activated following Cre-recombination. Such an approach allows to study ribosomal transcripts specifically active for the desired cre-driver (148). To gain more fundamental insights into expressed genes, their regulation in health and disease as well as more definite view into neuronal subpopulations, the readers are kindly encouraged to upload their sequencing datasets into public available bioportals. This will allow the scientific community to browse for their genes of interest. Future studies should especially focus on comparing steady state gene expression to different diseases to unravel important transcripts in certain neuronal subpopulations.

If we consider the central role of the nervous system in regulating immune functions, the potential is remarkable. The work of Tracey and colleagues has underlined its potential by the discovery of the ‘cholinergic anti-inflammatory’ pathway (143, 149). Such profound anti-inflammatory properties are of need for overwhelming inflammatory diseases, such as inflammatory bowel disease, sepsis, rheumatoid arthritis, and many more. Of note, the therapeutic potential of the anti-inflammatory properties of the nervous system has been successfully tested in clinical trials in humans (144, 150, 151). Even more impressive is the notion that patients in one clinical trial did no longer respond to conventional anti-inflammatory treatments, whereas vagal nerve stimulation improved clinical symptoms of rheumatoid arthritis patients (144). However, there is an emerging need to identify neuronal mechanisms that construct anti- as well as pro-inflammatory reactions in more detail. Such insights may uncover key neuro-immune cues that can potentially be harnessed by drugs or biologics in the near future.

## Author Contributions

MJ: writing of manuscript. MK-B: bioinformatic analysis and critical revision of the manuscript. DD: writing of the manuscript. SM: design of figures and critical revision of the manuscript. CK: writing of manuscript. All authors contributed to the article and approved the submitted version.

## Funding

This work was supported by grants from the Swiss National Science foundation (Grant-ID: 184425 to MJ), the German Research Foundation (DFG; Neuromac SFB TR project B17, FOR2599 project 5 - KL 2963/5-2, KL 2963/2-1 and KL 2963/3-1 to CK), (DFG; SPP1937-DI764/9 to MK-B), and the European Research Council Starting Grant (ERCEA; 803087 to CK).

## Conflict of Interest

The authors declare that the research was conducted in the absence of any commercial or financial relationships that could be construed as a potential conflict of interest.

## Supplementary Material

Supplementary Figure 1 Expression of chemokine receptors, C-type leptin receptors and other genes of interest in different ENS subsets. Dotplots showing the percentage of expressing cells as well as average expression within the indicated identified neuronal clusters for selected murine chemokine receptor, C-type leptin receptor and other genes of interest. Data was downloaded from http://mousebrain.org/ (12) or from https://singlecell.broadinstitute.org/ (14). Genes not present in the plot showing the Drokhlyanski data have been filtered. All dataset were normalized and transformed before the plots were created using the Seurat package (16).
