# Supplementary figures and images for: An Integrated View on Neuronal Subsets in the Peripheral Nervous System and Their Role in Immunoregulation
Source: Front Immunol. 2021 Jul 12;12:679055. doi: 10.3389/fimmu.2021.679055 (PMC8312561; doi:10.3389/fimmu.2021.679055)

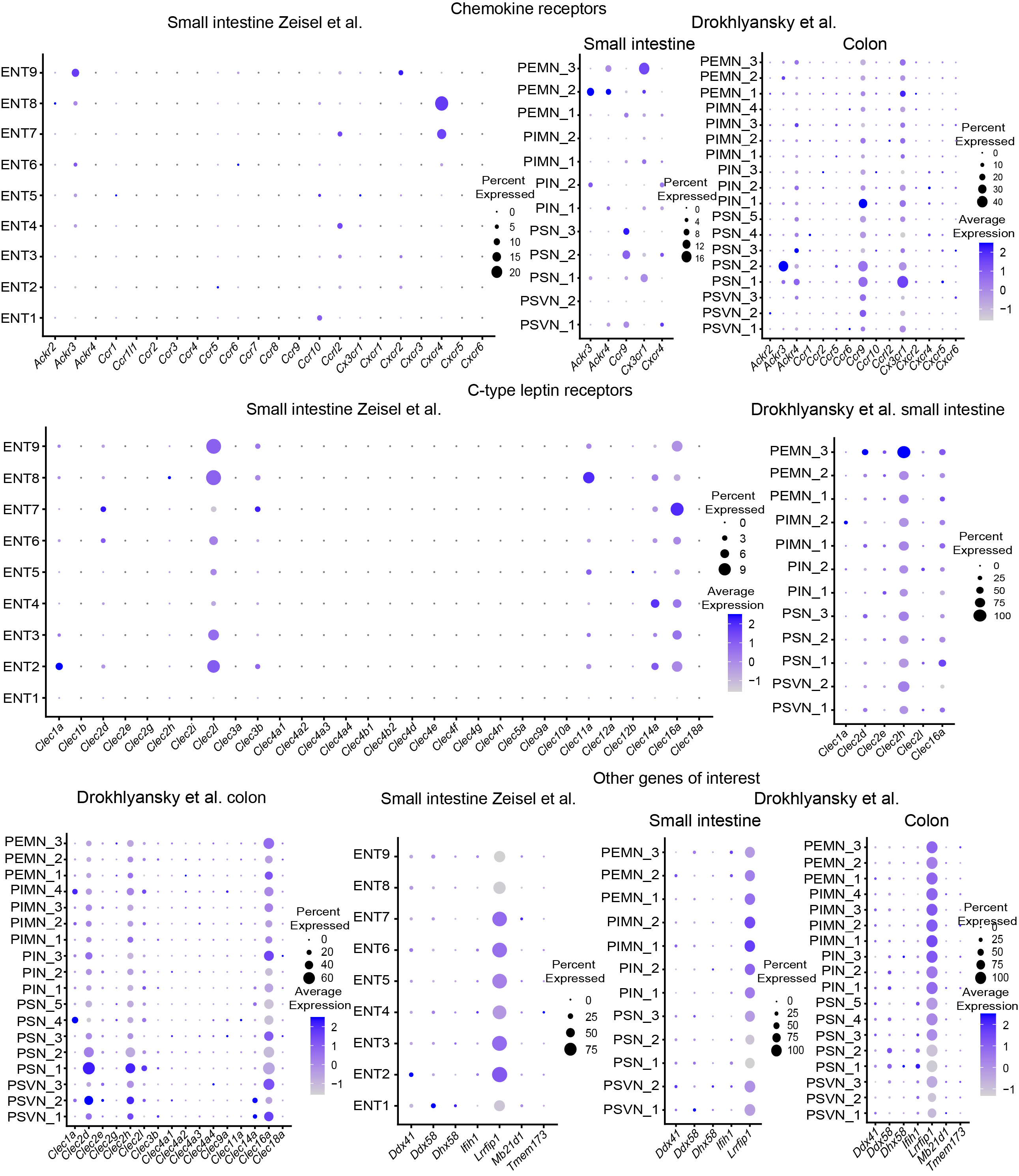

Supplement: Supplementary Figure 1 — Expression of chemokine receptors, C-type leptin receptors and other genes of interest in different ENS subsets. Dotplots showing the percentage of expressing cells as well as average expression within the indicated identified neuronal clusters for selected murine chemokine receptor, C-type leptin receptor and other genes of interest. Data was downloaded from http://mousebrain.org/ (12) or from https://singlecell.broadinstitute.org/ (14). Genes not present in the plot showing the Drokhlyanski data have been filtered. All dataset were normalized and transformed before the plots were created using the Seurat package (16). [file Image_1.jpg]
